# Italian validation of the short form of the Pelvic Organ Prolapse/Urinary Incontinence Sexual Questionnaire (PISQ-12)

**DOI:** 10.1007/s00192-022-05235-0

**Published:** 2022-06-01

**Authors:** Silvia Volonte’, Marta Barba, Alice Cola, Giuseppe Marino, Matteo Frigerio

**Affiliations:** ASST Monza, Ospedale San Gerardo, Via Pergolesi 33, Monza, Italy

**Keywords:** Pelvic floor disorders, Quality of life questionnaire, Sexual life, Pelvic organ prolapse, Urinary incontinence

## Abstract

**Introduction and hypothesis:**

The aim of this study was to translate the English short form of Pelvic Organ Prolapse/Urinary Incontinence Sexual Questionnaire (PISQ-12) and evaluate its validity, internal consistency, and test-retest reliability.

**Methods:**

The questionnaire was translated into Italian by standardized procedural steps, and the final version was submitted to women referred to urogynecological outpatient care for genital prolapse or urinary incontinence reporting sexual disorders (cases) or not (controls). For the test-retest evaluation, cases received the questionnaire 2 weeks later. The Wilcoxon test (non-parametric) was used to assess differences between cases and controls. Convergent validity was tested with the Italian version of the FSFI-19. The internal consistency was tested using Cronbachʼs alpha. The degree of concordance/agreement was measured with Cohenʼs kappa. The absolute agreement of test-retest results was tested with the intraclass correlation coefficient (ICC).

**Results:**

Sixty women were recruited for the study and answered the questionnaire. The overall rate of missing items was 1.3%. Construct validity was demonstrated, as the questionnaire discriminated significantly between patients with and without symptoms. Convergent validity with FSFI-19 was tested, and a linear correlation between scores was demonstrated (F < 0.001). Internal consistency reliability evaluated with Cronbach's alpha was satisfactory (0.54–0.81). Cohenʼs kappa values as absolute agreement coefficients were between 0.59 and 0.80 (good agreement). Intraclass correlation coefficients ranged between 0.88 and 0.94 (very satisfactory agreement) for each functional domain.

**Conclusions:**

The Italian version of the PISQ-12 is reliable, valid, and consistent.

**Supplementary Information:**

The online version contains supplementary material available at 10.1007/s00192-022-05235-0.

## Introduction

Quality of life (QoL) assessment is a milestone of clinical practice in gynecology. In particular, the use of validated QoL questionnaires is of the utmost importance for pelvic floor disorders because of their functional nature and high prevalence. Since pelvic floor disorders are often associated with each other, women with genital prolapse or urinary incontinence may have a particularly high prevalence of sexual disorders [1]. Validated QoL questionnaires allow the assessment of sexual domain symptom frequency and severity, their impact on quality of life, and trends over time. Moreover, self-completed questionnaires are preferable to clinical interviews as they minimize bias related to the caregiver. Lastly, a structured questionnaire that covers all sexual life aspects would be a useful tool to screen this population. Although several sexual life QoL questionnaires are available in the general population, there are very few questionnaires specifically designed for women with genital prolapse and urinary incontinence. One of them is represented by the FSFI-19, a 5-point Likert scale self-reported questionnaire with 19 items covering six domains of sexual function (sexual desire, lubrication, arousal, orgasm, pain, and satisfaction). An Italian version of this questionnaire is available and can be used to evaluate sexual dysfunction in very different conditions [2]. However, since sexual dysfunction represents one of the traditional four domains of pelvic floor disorders (bladder, bowel, prolapse, sex), which may often overlap each other, it would be very interesting to have more specific tools available [3]. These are represented by the Pelvic Organ Prolapse/Urinary Incontinence Sexual Questionnaire (PISQ-31) and its short form (PISQ-12), which are specifically designed to investigate sexual function in women with pelvic organ prolapse (POP) and urinary incontinence (UI) [4]. In particular, PISQ-12 may represent a very interesting tool because of its easy handling and brevity. Moreover, the PISQ-12 questionnaire also involves the evaluation of partner sexual function with a specifically designed domain, which clearly has a crucial role in couples’ sexual well-being. Unfortunately, this questionnaire has not been validated in the Italian language yet.

Consequently, the aim of this study was to translate the short form of the Pelvic Organ Prolapse/Urinary Incontinence Sexual Questionnaire (PISQ-12) into the Italian language [4] and evaluate its validity, internal consistency, and test-retest reliability. This ensures that the questionnaire makes sense to patients, can differentiate between symptomatic patients and controls, and is able to measure what it was intended to measure and that the answer to each question will not change substantially if the questionnaire is administered twice over a short period.

## Methods

The study was conducted in San Gerardo Hospital, Monza, Italy. IRB (Ethics Committee of San Gerardo Hospital) approval was obtained before starting the study. The considered questionnaire (PISQ-12) represents a short form derived from the Pelvic Organ Prolapse/Urinary Incontinence Sexual Questionnaire (PISQ-31). The PISQ-12 is a validated 12-item questionnaire for sexual life assessment in patients who suffer from urinary incontinence or pelvic organ prolapse [4]. The PISQ-12 is a self-administered questionnaire evaluating sexual function in three domains: behavioral-emotive (items 1–4), physical (items 5–9), and partner-related (items 10–12). Answers are graded on a 5-point Likert scale ranging from 0 (always) to 4 (never), with the first four items using a reverse score. The total score ranges from 0 (worst possible sexual function) to 48 (best possible sexual function). In the case of missing items (up to 2 missing items allowed), the total score is calculated by multiplying the mean of the responses by the number of items.

### Translation

The validation process of a linguistic translation must maintain conceptual and technical equivalence between the source and target language [5]. The questionnaire was translated into Italian by the following procedural steps [6]. A preliminary translation from English into Italian was carried out in parallel by two native Italian-speaking translators, with English as their first foreign language. Then, a consensus meeting among translators and the research group was held to compare the two Italian versions and yielded a first consensus Italian version of the questionnaire. After that, a native English-speaking translator with Italian as his first foreign language back-translated the Italian consensus version. A second consensus meeting was held between the English mother-tongue translator and clinical investigators, during which the back-translated and original questionnaires were compared and differences discussed. The process led to a revised version of the first consensus questionnaire. The comprehension of the obtained Italian consensus version was therefore tested in a real-life population to assess questionnaire comprehension. The questionnaire was submitted to a group of ten women referred to urogynecology outpatient clinic for pelvic organ prolapse or urinary incontinence, after standard evaluation including obstetric and gynecological history collection, medical interview, and urogynecological examination, as previously described [7]. They were asked to evaluate their perceived degree of difficulty in understanding each question item, and unstructured feedback was collected. All women correctly understood questions and precoded answers and no item was therefore changed. After that, the final Italian version of the questionnaire was obtained (Supplemental Material 1).

### Study participants

Gynecological outpatients of San Gerardo Hospital, Monza, Italy, were recruited. In women referred to urogynecology outpatient care, obstetric and gynecological history was collected, a medical interview assessing pelvic floor disorder-related symptoms and sexual activity was undertaken, and a urogynecological examination was performed, as previously described [7]. Women referred to urogynecological outpatient care for genital prolapse or urinary incontinence whose mother tongue was Italian, aged 18 years and over, were included. Exclusion criteria included insufficient Italian language proficiency, sexual inactivity, and psychiatric or neurological disorders. The leading symptom requiring the urogynecological consult, as explained by the patient during the clinical interview (prolapse symptoms, urinary incontinence, or both), was noted. Study participants completed the questionnaire during clinical interviews. The questionnaire was submitted to women reporting sexual disorders (cases) and to asymptomatic patients (controls), after obtaining informed written consent from each study participant. Cases and controls were defined, as done previously, with respect to sexual symptoms using the question: “How much do your sexual symptoms bother you?” and the following choice of answers: “I do not have symptoms,” “not at all,” “a little,” “quite a lot,” and “very much” [8]. Controls were identified as women answering “I do not have symptoms” or “not at all;” otherwise patients were defined as cases. For the test-retest evaluation, cases received the questionnaire 2 weeks later by email. Questionnaire distribution and all interviews were undertaken by the authors.

### Questionnaire validation

Construct validity was tested to guarantee that the questionnaire is able to discriminate between women with and without pelvic floor symptoms [9]. To test validity, the questionnaire was administered to women with and without sexual disorders (respectively defined as ‘cases' and ‘controls'). Total scores for women with and without significant symptoms were compared and tested for statistical differences to assess validity. Given the heterogeneity of variances, the Wilcoxon test (non-parametric) was used to assess differences between cases and controls. Convergent validity was tested with the Italian version of the FSFI-19. The internal consistency—the strength of association among items—was tested using Cronbachʼs alpha [10, 11]. The test-retest reliability analysis was aimed to determine the questionnaire’s reproducibility over time by giving the questionnaire at baseline and 2 weeks later [6]. The degree of concordance/agreement was measured with Cohenʼs kappa [12]. In addition, the absolute agreement of test-retest results of different individuals was tested with the intraclass correlation coefficient (ICC) [13, 14].

Statistical analysis was performed with JMP 7.0 (SAS, Cary, NC, USA). Where ratings were missing, items were excluded from the analysis pool. Patients who did not complete the questionnaire at both baseline and the test-retest visit were excluded from the analyses. *P* < 0.05 was considered as significant.

## Results

**Fig. 1.**
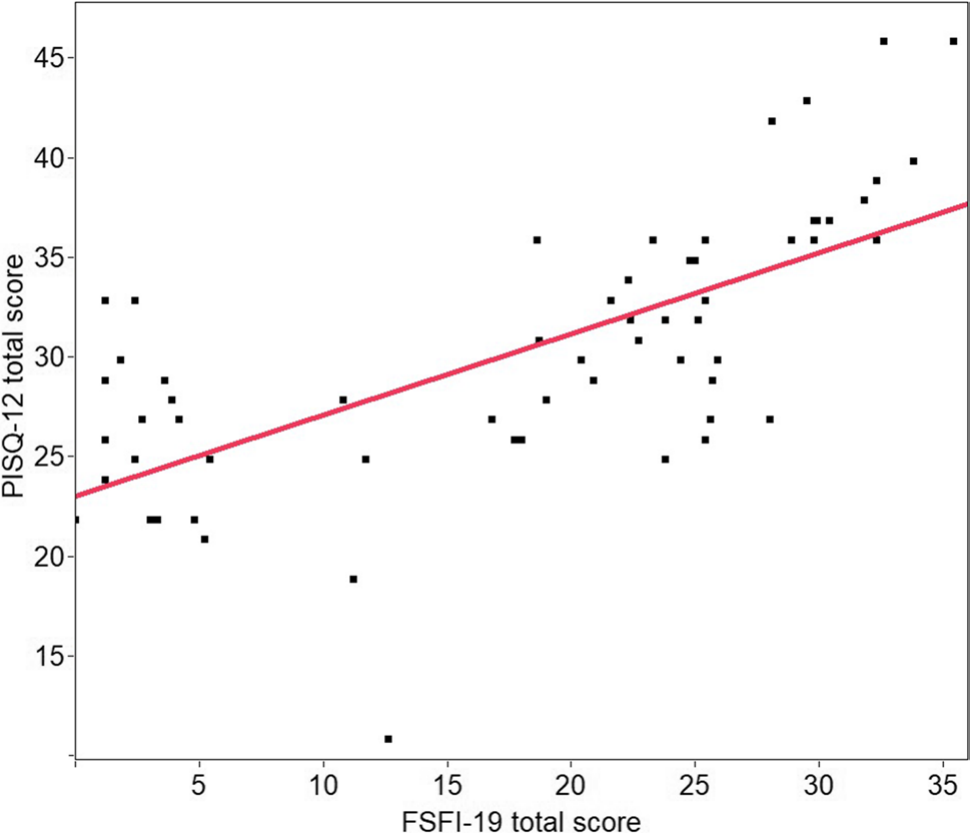
Convergent validity; F < 0.001

In total 60 women answered the questionnaire. Population characteristics are shown in Table 1. There was no dropout since all of them completed the questionnaire. Overall rate of missing items was 1.3%. Based on the self-reported presence of significant sexual symptoms to the question “How much do your sexual symptoms bother you?,” the population was divided into 41 cases and 19 controls. Construct validity was demonstrated, as the questionnaire discriminated significantly between patients with and without symptoms (Table 2). Specifically, patients who were symptomatic in any domain reached a score at least one point higher than asymptomatic women, which corresponds to the minimal important difference, as previously established [12]. Convergent validity with FSFI-19 was tested, and a linear correlation between scores was demonstrated (F < 0.001; Fig. 1). Internal consistency reliability evaluated with Cronbach's alpha was 0.77 for the whole questionnaire. Moreover, Cronbach’s alpha scored 0.81, 0.64, and 0.54 for behavioral-emotive, physical, and partner domains, respectively (range 0.54–0.81; Table 3). Test-retest reliability evaluation is shown in Table 3. Cohenʼs kappa values as absolute agreement coefficient were between 0.59 and 0.80, showing overall good agreement. Specific Cohen's kappa for each item is reported in Supplementary Material 2. Intraclass correlation coefficients ranged between 0.88 and 0.94, indicating a very satisfactory overall agreement for each functional domain.

**Table 1:** Population characteristics. PFD = pelvic floor disorder; POP = pelvic organ prolapse; UI = urinary incontinence

Age (years)	57.4 ± 8.6
Sexual disorders (cases)	41 (68.3%)
POP	42 (70.0%)
UI	27 (45.0%)

**Table 2:** Construct validity assessment

Domain	Cases	Controls	*P* value
Behavioral-emotive	7.9 ± 3.0	9.8 ± 3.4	0.047
Physical	13.0 ± 3.3	18.1 ± 2.3	< 0.001
Partner	7.0 ± 1.4	8.7 ± 1.9	0.002
Total score	27.9 ± 4.9	36.5 ± 56.1	< 0.001

**Table 3:** Test-retest reliability. Cohen’s kappa values and intraclass correlation coefficients (ICCs) for each domain. Cohen’s kappa values are reported as ranges for the three functional domains

Domain	Cohen’s kappa range	Domain score ICC	*P* value
Behavioral-emotive domain	0.63–0.79	0.88	< 0.001
Physical domain	0.66–0.76	0.94	< 0.001
Partner domain	0.59–0.80	0.89	< 0.001

## Discussion

According to a very recent systematic census of Italian validated questionnaires on pelvic floor disorders, four questionnaires for sexual dysfunction are available, namely the McCoy Female Sexuality Questionnaire, Sexual Quality of Life-Female (SQOL-F), Positive Sexuality Scale (PSS), and FSFI-19 [15]. However, none of them has been specifically designed and validated in women with pelvic organ prolapse and urinary incontinence like the PISQ-12. In the present study, we translated and tested the validity of the Italian version of the questionnaire. Translation and linguistic validation of a QOL questionnaire are important and should be implemented before the questionnaire is used in the clinical setting and in research in a population that speaks a different language so that a unified standard guide for assessing a disease can be achieved [16]. A questionnaire that is valid and reliable for a particular language may not be valid and reliable when used in a different population and could fail to reproduce the same findings when used in other scenarios and by other observers [17].

No issues arose from the translation process, which was carried out following the method proposed by Guillemin, which consisted of a forward and backward translation plus researcher-translator consensus meetings [6]. The obtained version of the questionnaire was tested for comprehension—according to the widely accepted process for linguistic validation [18]—and resulted in no difficulties in understanding each question item and related pre-coded answers in a real-life population. Construct validity was confirmed, as the questionnaire was able to discriminate between patients with and without symptoms for the three domains of the questionnaire. Convergent validity was tested and demonstrated with FSFI-19. The association of individual items in each domain was evaluated with the internal consistency reliability analysis using Cronbach’s alpha. Lastly, the longitudinal stability of the questionnaire was evaluated and confirmed with test-retest reliability and intraclass correlation coefficient analysis.

The PISQ-12 has some theoretical advantages compared to FSFI-19. The latter is a 19-item self-report measure of female sexual function that provides scores on overall levels of sexual function, including sexual desire, arousal, orgasm, pain, and satisfaction [19]. Among these, one of the weak points is represented by the pain domain, which is expressly investigated in terms of “penetration” rather than “sexual activity,” given that sexual pain almost always occurs in response to vaginal insertion of some kind (i.e., penile, digital, etc.) [20]. On the opposite side, this aspect is described far more generally in the PISQ-12, in which the pain domain could be connected to different aspects of sexual activity, thus reducing the risk of underestimating this symptom. Another strength of the PISQ-12 is represented by the specific questions addressed to investigate male partners’ sexual dysfunctions and their potential negative impact on sexual intercourses, which is missing in the FSFI-19. Moreover, the PISQ-12 is considerably shorter than the FSFI-19, and this can result in an easier and more intuitive application, especially when the questionnaire needs to be filled in in a short time or by an advanced age population such as the one most affected by PFDs [21]. Another possible criticism of FSFI-19 is that sexual function is evaluated over the past 4 weeks [20]. Since the PISQ-12 evaluates the last 6 months, this reduces the risk of misjudging sexual disorders and consequently can be considered a more reliable tool to investigate sexual function. Lastly, general questionnaires focused on sexual function such as the FSFI, which underwent validation and reliability testing in a general population, may not be sensitive enough to detect differences that are associated with other pelvic floor dysfunctions. This can be very relevant, considering that specific PFDs—such as different types of urinary incontinence—have been demonstrated to have a particularly detrimental impact on women’s sexual function [1]. On the contrary, the PISQ-12 is a condition-specific questionnaire focused on sexual function for use in women with specific PFDs and has undergone rigorous validation and reliability testing [4]. This may be particularly relevant; since prolapse can result in dramatic changes to the urogenital tract, it is a hidden disfigurement that only the woman and her intimate contacts are aware of and may negatively affect a woman’s body image. Similar consideration can be made for urinary incontinence, which can severely affect sexual well-being. These conditions can lead to shame, embarrassment, and a decrease in feelings of sexual attractiveness that may negatively impact her quality of life [22].

Strengths of the study include standardized procedural steps for translation/validation and its originality, being that—to our knowledge—the questionnaire is the first to specifically evaluate sexual function in women with pelvic floor disorders in the Italian language and to evaluate the test-retest reliability. The main limitation of the study is the small sample size. In conclusion, the present study demonstrated good results for the Italian version of the PISQ-12 questionnaire. A validated Italian questionnaire is now available for clinical use to investigate the incidence, severity, and impact on the quality of life of sexual symptoms in women with pelvic floor disorders.

## Conclusions

In conclusion, this study demonstrated that the Italian version of the PISQ-12 is reliable, valid, and consistent. A validated questionnaire is now available to investigate the incidence, severity, and impact on QoL of sexual symptoms in patients with urinary incontinence and pelvic organ prolapse. This study also confirms that sexual function is commonly affected by urogynecological symptoms and should be assessed in women with pelvic floor disorders.

## Supplementary Information

Supplementary Material 1. Italian version of PISQ-12 (PDF 80 kb)

Supplementary Material 2. Cohen’s kappa values for single items (PDF 45 kb)
