# Supplementary material for: Italian validation of the short form of the Pelvic Organ Prolapse/Urinary Incontinence Sexual Questionnaire (PISQ-12)
Source: Int Urogynecol J. 2022 Jun 1;33(11):3171–5. doi: 10.1007/s00192-022-05235-0 (PMC9569287; doi:10.1007/s00192-022-05235-0)
Supplement: Supplementary file 1 — Italian version of PISQ-12 (PDF 80 kb) [file 192_2022_5235_MOESM1_ESM.pdf]

PISQ-12 Italiano

Istruzioni: a seguire troverà una serie di domande sulla vita sessuale sua e del suo partner. Tutte le informazioni sono strettamente confidenziali. Le risposte saranno utilizzate per aiutare il medico a capire quali aspetti risultano importanti per la vita sessuale del paziente. La preghiamo di mettere la crocetta nel riquadro che meglio descrive la sua situazione. Nel rispondere alle domande consideri la sua vita sessuale degli ultimi 6 mesi. Grazie per l'aiuto.

1) Quanto spesso prova desiderio sessuale? Questo può includere la volontà di fare sesso, la pianificazione di fare sesso, la frustrazione derivante dalla mancanze di sesso, ecc.
□ *sempre* □ *spesso* □ *qualche volta* □ *raramente* □ *mai*

2) Raggiunge l'orgasmo quando ha rapporti sessuali col partner?
□ *sempre* □ *spesso* □ *qualche volta* □ *raramente* □ *mai*

3) Prova eccitazione sessuale durante l'attività sessuale col partner?
□ *sempre* □ *spesso* □ *qualche volta* □ *raramente* □ *mai*

4) Quanto è soddisfatta della varietà presente nella sua attuale vita sessuale?
□ *sempre* □ *spesso* □ *qualche volta* □ *raramente* □ *mai*

5) Sente dolore durante i rapporti sessuali?
□ *sempre* □ *spesso* □ *qualche volta* □ *raramente* □ *mai*

6) E' incontinente alle urine (perde urine) durante l'attività sessuale?
□ *sempre* □ *spesso* □ *qualche volta* □ *raramente* □ *mai*

7) La paura di episodi di incontinenza (feci o urine) limita la sua attività sessuale?
□ *sempre* □ *spesso* □ *qualche volta* □ *raramente* □ *mai*

8) Evita i rapporti sessuali a causa della sensazione di avere un corpo estraneo in vagina (vescica, retto, o vagina che scendono?)?
□ *sempre* □ *spesso* □ *qualche volta* □ *raramente* □ *mai*

9) Quando ha rapporti sessuali con il partner, prova emozioni negative come paura, disgusto, vergogna o colpa?
□ *sempre* □ *spesso* □ *qualche volta* □ *raramente* □ *mai*

10) Il tuo partner ha problemi di erezione che influenzano negativamente la sua attività sessuale?
□ *sempre* □ *spesso* □ *qualche volta* □ *raramente* □ *mai*

11) Il tuo partner ha problemi di eiaculazione precoce che influenzano negativamente la sua attività sessuale?
□ *sempre* □ *spesso* □ *qualche volta* □ *raramente* □ *mai*

12) Rispetto agli orgasmi che provava in passato, quanto intensi sono stati gli orgasmi che ha avuto negli ultimi sei mesi?
□ *molto meno intensi* □ *meno intensi* □ *stessa intensità* □ *più intensi* □ *molto più intensità*

<u>Punteggi</u>

I punteggi sono calcolati sommando i valori di ogni risposta con 0=mai e 4=sempre. Un punteggio inverso è invece attribuito alle domande 1, 2, 3 e 4. Il questionario breve può contemplare fino a due domande senza risposta. Per gestire eventuali risposte mancanti, il totale si calcola moltiplicando la media delle risposte ottenute per il numero di item. Se ci sono più di due risposte mancanti, la forma breve non è più in grado di prevedere il punteggio del questionario in forma estesa con buona approssimazione. I punteggi della forma breve possono solo essere riportati come

totale o per singolo quesito. Per confrontare i punteggi della forma breve e della forma estesa è necessario moltiplicare il primo per 2.58 (31/12).
