# Supplementary material for: Italian validation of the short form of the Pelvic Organ Prolapse/Urinary Incontinence Sexual Questionnaire (PISQ-12)
Source: Int Urogynecol J. 2022 Jun 1;33(11):3171–5. doi: 10.1007/s00192-022-05235-0 (PMC9569287; doi:10.1007/s00192-022-05235-0)
Supplement: Supplementary file 2 — Cohen’s kappa values for single items (PDF 45 kb) [file 192_2022_5235_MOESM2_ESM.pdf]

Supplementary Material 2

Cohen's kappa values for single items

#1 (behavioral-emotive domain)	0.63
#2 (behavioral-emotive domain)	0.79
#3 (behavioral-emotive domain)	0.71
#4 (behavioral-emotive domain)	0.63
#5 (physical domain)	0.68
#6 (physical domain)	0.76
#7 (physical domain)	0.66
#8 (physical domain)	0.72
#9 (physical domain)	0.69
#10 (partner-related domain)	0.80
#11 (partner-related domain)	0.63
#12 (partner-related domain)	0.59
